# Fatty Acid-and Retinol-Binding Protein, Mj-FAR-1 Induces Tomato Host Susceptibility to Root-Knot Nematodes

**DOI:** 10.1371/journal.pone.0064586

**Published:** 2013-05-22

**Authors:** Ionit Iberkleid, Paulo Vieira, Janice de Almeida Engler, Kalia Firester, Yitzhak Spiegel, Sigal Brown Horowitz

**Affiliations:** 1 Department of Entomology, Nematology and Chemistry units; Agricultural Research Organization (ARO), the Volcani Center, Bet Dagan, Israel; 2 Department of Plant Pathology and Microbiology, the Faculty of Agriculture Food & Environment, the Hebrew University of Jerusalem, Rehovot, Israel; 3 UMR Institut Sophia Agrobiotech INRA/CNRS/UNS, Sophia Antipolis, France; 4 NemaLab/ICAAM – Instituto de Ciências Agrárias e Ambientais Mediterrânicas, Universidade de Évora, Évora, Portugal; Volcani Center, Israel

## Abstract

Plant-parasitic nematodes produce at least one structurally unique class of small helix-rich retinol- and fatty-acid-binding proteins that have no counterparts in their plant hosts. Herein we describe a protein of the plant-parasitic root-knot nematode *Meloidogyne javanica*, which is a member of the nematode-specific fatty-acid- and retinol-binding (Mj-FAR-1) family of proteins. The *mj-far*-*1* mRNA was detected through *M. javanica* pre-parasitic J2s, migratory and sedentary parasitic stages by quantitative reverse transcriptase polymerase chain reaction (qRT-PCR). Immunolocalization assays demonstrate that the FAR protein of *Meloidogyne* is secreted during sedentary stages, as evidenced by the accumulation of FAR at the nematode cuticle surface and along the adjacent host root tissues. Tomato roots constitutively expressing *mj-far*-*1* demonstrated an increased susceptibility to root-knot nematodes infection as observed by accelerated gall induction and expansion, accompanied by a higher percentage of nematodes developing into mature females compared to control roots. RNA interference assays that expressed double-stranded RNA complementary to *mj-far-1* in transgenic tomato lines specifically reduced nematode infection levels. Histological analysis of nematode-infested roots indicated that in roots overexpressing *mj-far*-*1*, galls contained larger feeding cells and might support a faster nematode development and maturation. Roots overexpressing *mj-far*-*1* suppressed jasmonic acid responsive genes such as the proteinase inhibitor (*Pin2*) and γ*-thionin*, illustrating the possible role of Mj-FAR-1 in manipulating the lipid based signaling *in planta*. This data, suggests that *Meloidogyne* FAR might have a strategic function during the interaction of the nematode with its plant host. Our study present the first demonstration of an *in planta* functional characterization and localization of FAR proteins secreted by plant-parasitic nematodes. It provides evidence that Mj-FAR-1 facilitates infection most likely via the manipulation of host lipid-based defenses, as critical components for a successful parasitism by plant-parasitic nematodes.

## Introduction

Plant-parasitic nematodes (PPN) represent one of the largest sources of biotic stress experienced by plants and are difficult to control. Among the most devastating PPN are the sedentary root-knot nematodes (RKN), *Meloidogyne* spp., which are obligate biotrophs [1]. As sedentary parasites, their development is strictly dependent on nematode feeding site (NFS) formation and maintenance, ensuring the continuous nutrient supply for the juvenile nematode until maturation [2]. Although not much is known of how RKN establish their giant-feeding cells (GCs) most studies suggest that nematode glandular secretions (so called effectors) injected into plant cells by these nematodes interact directly or indirectly with plant components, leading to the establishment and maintenance of NFSs [2]–[7].

During the last decades extensive genome, transcriptome and proteome studies have shown that many of these effectors are synthesized in three specialized esophageal glands [8]–[11]. Other organs such as amphids and cuticle that are in permanent contact with the environment also produce secretory proteins. To date, several cuticle proteins from PPN have been identified, which the functional characterization suggests their importance for parasitism [12]–[14].

As obligate endoparasites that complete most of their life-cycle within plant roots, the RKN's ability to overcome plant defense mechanisms and maintenance of their feeding cells is reliant on continuous suppression of plant defenses [15], [16]. Among components of the plant defense signaling cascades, fatty acid (FA) metabolic pathways mediated via lipid signaling molecules, known as oxylipins, regulate various defense and developmental pathways. These, FA metabolic pathways, also present unique signaling elements shared among plants and vertebrates [17]. Being catalyzed by the activities of the lipoxygenase (LOX: 9-LOX or 13-LOX) and α-dioxygenase enzyme families [17], [18], oxylipins are specifically induced upon pathogen infection [19]–[24]. Jasmonic acid (JA) is one of the best-characterized oxylipins, that is produced enzymatically and accumulate in response to various stresses, particularly to wounding and pathogen infection [25]. Previous studies suggest that hormone signaling pathways may be manipulated by nematodes during the initiation and maintenance of their feeding sites [26]–[33]. Thus, it is likely that hormone balance manipulation is mediated by nematode's secreted effector proteins.

Secreted fatty acid and retinol binding proteins (FAR) have been proposed to facilitate animal-parasitic nematodes infection by scavenging and transporting fatty acids, required for developmental processes and cellular differentiation of the parasite [34]–[38]. FAR proteins may also play a role in interfering with intracellular and intercellular lipid signaling related to host defenses [34]–[40]. Up till now, extensive studies were carried on FAR of animal and human parasites, particularly in the temporal and spatial transcription and fatty acid binding characteristics [34]–[38], [41]. Analysis the FAR protein (Gp-FAR-1) of the cyst nematode *Globodera pallida* was the first study provided for PPN [40]. Being localized to the cuticle surface of the pre-parasitic J2 of this nematode species, Gp-FAR-1 has shown to interfere with the plant LOX-mediated defense signaling by binding to LOX substrates and products [40]. Although investigation on ligand binding properties of recombinant FAR proteins have been reported, the *in vivo* role of FAR proteins remains to be shown [36], [39], [40].

Herein we identified and investigated the function of the first RKN FAR protein (Mj-FAR-1) during the interaction of *M. javanica* and its tomato host. This RKN fatty acid and retinol-binding protein (FAR) is being transcribed from early pre-parasitic to sedentary stages. We also show for the first time that FAR is secreted into the surrounding host tissues and might play a role in promoting nematode parasitism, and thus participating in modulating host susceptibility to PPN.

## Results

### *mj-far-1* identification and sequence homology

**Figure 1:**
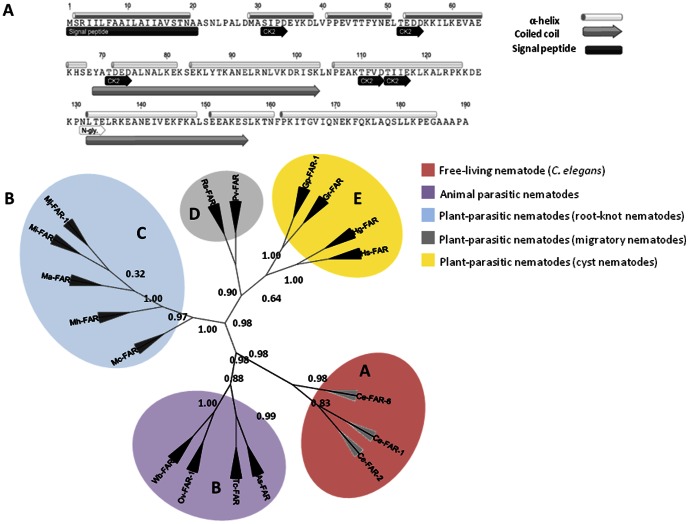
Mj-FAR-1 characterization. **A.** Primary and secondary structure analysis of predicted Mj-FAR-1. A putative hydrophobic leader/signal peptide, as predicted by the SignalP program is shown at the N terminus along with a cleavage site between two alanine residues at position 20–21. Consensus *N*-linked glycosylation site (N-gly) and the consensus casein kinase II phosphorylation sites (CK2) are indicated. Secondary structure analysis through Jpred predicts a predominantly alpha-helical conformation (gray cylinder) with coiled-coil structures (gray arrow) as shown. **B.** Phylogenetic tree of Mj-FAR-1 with other nematode FAR proteins. The tree was constructed with FAR protein sequences of: *C. elegans* (Ce-FAR-1, Ce-FAR-2 and Ce-FAR-6) belong to group A, human parasitic nematodes *O. volvulus* (Ov-FAR-1) and *W. bancrofti* (Wb-FAR), animal parasitic nematodes *A. suum* (As-FAR) and *T. canis* (Tc-FAR), and plant-parasitic nematodes, *M. javanica* (Mj-FAR-1), *M. incognita* (Mi-FAR), *M. arenaria* (Ma-FAR), *M. hapla* (Mh-FAR) and *M. chitwoodi* (Mc-FAR), cyst forming nematodes *G. pallida* (Gp-FAR-1), *G. rostochiensis* (Gr-FAR), *H. glycines* (Hg-FAR), *H. schachtii* (Hs-FAR) and migratory endoparasites *P. vulnus* (Pv-FAR) and *R. similis* (Rs-FAR). Bootstrap values are shown at each node. 1,000 bootstrap replicates were obtained, with nearly the same results, and only a single tree retrieved from the phylogenetic relationship analysis is shown. Five clusters in the phylogenetic tree have been arbitrarily assigned the names A, B, C, D and E.

BLAST searches against *M. javanica* ESTs database (www.nematode.net; www.nematodes.org) with the *G. pallida* Gp-FAR-1 (accession no. CAA70477.2) revealed the presence of one cDNA's encoding a FAR homolog, designated herein as *mj-far-1* (DNA) or Mj-FAR-1 (protein), following the previously suggested nomenclature convention for this group of proteins [42]. Further extension of *mj-far*-*1* by genome walking identified a 802 bp fragment encompassing an open reading frame (ORF), encoding a predicted Mj-FAR-1 protein of 190 aa with a deduced molecular mass of 21.2 kDa and a theoretical pI 5.72. *In silico* analysis of the deduced Mj-FAR-1 amino acid sequence through SignalP 3.0 [43], PSORTII [44], and TargetP [45] algorithms indicate the presence of twenty amino acids comprising an hydrophobic signal peptide. This signal peptide contains a predicted cleavage site located between position 20 (alanine) and 21 (alanine), directing the protein to the secretory system, a characteristic of PPN's parasitism gene products [8], [46] (Fig. 1A). Secondary structure analysis of Mj-FAR-1 was conducted by PHD [47]; http://www.predictprotein.org) and Jpred [48]; http://www.compbio.dundee.ac.uk/www-jpred/) algorithms. These analyses revealed that in common with others FARs of animal and plant parasites [38], [40] a predominantly alpha-helical conformation is predicted containing a 81.6% helix, 18.4% loop and no beta/extended structure was identified (Fig. 1A). In addition, the observed coiled-coil structure between positions 60±100 and 120±160 [49]; http://www.ch.embnet.org/software/COILS_form.html), was another common structural property shared with other parasite origin FAR. Likewise, in common with all other counterparts of this protein, a N-linked glycosylation site (Asn-Leu-Thr-Glu) presented at residues 131 along with conserved consensus casein kinase II phosphorylation sites at residues 31, 52, 71, 110 and 114 predicted by the PHD [47] algorithms were observed [38] (Fig. 1A). To study the phylogenetic relationship among the predicted amino acid sequence of Mj-FAR-1 and other nematodes FAR, a BLASTp search through available online databases (www.nematode.net; www.nematodes.org and www.ncbi.nlm.nih.gov) was conducted using the amino acid sequence of Gp-FAR-1 of *G. pallida* as the query using ClustalW in MEGA5.0 software (http://www.megasoftware.net/mega.php). A phylogenetic inference of the FAR proteins is presented in Fig. 1B, showing five main clusters A to E, represented by: FAR proteins of the free-living nematode *Caenorhabditis elegans* (clade A), animal-parasitic nematodes (clade B), root-knot nematodes (clade C), endo-migratory plant-parasitic nematodes (clade D) and cyst nematodes (clade E). Protein alignment of Mj-FAR-1 against Ce-FAR members of clade A determined a 41%, 39% and 31% sequence identity to Ce-FAR-1 (F02A9.2), Ce-FAR-2 (F02A9.3) and Ce-FAR-6 (W02A2.2) of *C. elegans*, respectively. As expected, Mj-FAR-1 grouped closely with those FARs belong to other parasitic nematodes, exhibiting 45%, 52%, 46% and 47% sequence identity with FARs of animal-parasitic nematodes (Clade B), such as *Ascaris suum* As-FAR (AS03229), *Toxocara canis* Tc-FAR (TCP00411_1), including the human-parasitic nematodes *Onchocera volvulus* Ov-FAR-1 (Q25619.1) and *Wuchereria bancrofti* Wb-FAR (WBP02641_1), respectively. The sequence similarity observed against cyst nematode species, represented by *G. pallida* Gp-FAR-1 (CAA70477.2), *G. rostochiensis* Gr-FAR (GRP01719_1), *Heterodera glycines* Hg-FAR (HG03356) and *H. schachtii* Hs-FAR (HS00476), vary between 64% to 68% against Mj-FAR-1. The sequence identity observed between Mj-FAR-1 and the FAR of migratory endoparasites nematodes increased from 73% to 75%, related to *Pratylenchus vulnus* Pv-FAR (PVP01247_1) and *Radopholus similis* Rs-FAR (RSP00855_1), respectively. The highest predicted amino acid identity were observed among *Meloidogyne* spp., with 98% sequence identity between Mj-FAR-1 and *M. incognita* Mi-FAR (MI02617) and *M. arenaria* Ma-FAR (MA00545), and 90% identity with *M. hapla* Mh-FAR (MH02761). Interestingly, the sequence identity between Mj-FAR-1 and *M. chitwoodi* Mc-FAR (MC02639) presented the lowest value of 74%.

### Expression pattern of *mj-far-1* gene during nematode development

**Figure 2:**
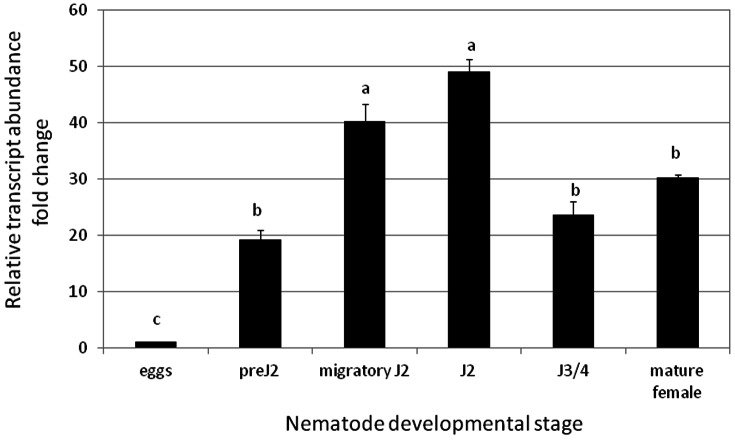
Kinetics of *mj-far*-1 gene transcripts through *M.* *javanica* life cycle stages. *mj-far-1* expression was detected for non-parasitic stages: eggs and pre-parasitic infective juvenile J2s; and within tomato roots, for migratory parasitic J2s (6 h and 48 h after infection), J2s and J3/J4 juveniles, and mature females (15 and 28 DAI, respectively). *mj-far*-*1* transcripts were normalized using the normalisation factor calculated as the geometric mean of the expression levels of two most stable nematode endogenous reference genes, *18S* and *EF-1α*. Each reaction was performed in triplicate and the results represented the mean of two independent biological replicates. Data represent the mean relative expression and standard error obtained from two independent biological experiments. Different letters above the bars denote a significant difference (*P≤*0.05, analysis of variance) between samples as analyzed by Tukey-Kramer multiple comparison test. The experiment was repeated three times and similar results were obtained.

The expression dynamics of *mj-far*-*1* of *M. javanica* for non-parasitic and parasitic stages was quantified using quantitative real-time reverse transcription-PCR (qRT-PCR). To assess the *mj-far-1* gene expression, equivalent RNA amounts were analyzed for different nematode stages: eggs and pre-parasitic J2s; and within tomato roots, the migratory parasitic J2s (6 h and 48 h after infection), J2s and J3/J4 juveniles (15 DAI) and mature females (28 DAI). RNA extracted from non-infected roots was used as a negative control; while transcript levels were normalized for each sample with the geometric mean of the expression of two selected housekeeping genes *18S* rRNA and EF-1α (Fig. 2). The lowest level of *mj-far*-*1* transcripts were detected within eggs, followed by a marked increase of 18 fold within pre-parasitic J2s (Fig. 2), suggesting a strong induction of *mj-far-1* transcripts just before the infection process of freshly hatched pre-parasitic J2s. Within the first hours after root infection a relative increase in *mj-far-1* transcripts was consistently observed in nematode juveniles collected at 6 h to 48 h after infection (Fig. 2). Expression level of *mj-far*-*1* in sedentary juveniles (J2 and J3/J4) collected at 15 DAI, and in adult females (28 DAI), showed some reduction during nematode development. The detectable levels of this gene transcripts throughout infection suggest a potential role of Mj-FAR during the different parasitic sedentary stages.

### FAR protein is secreted by root-knot nematodes and accumulates at the nematode-host cell interface

**Figure 3:**
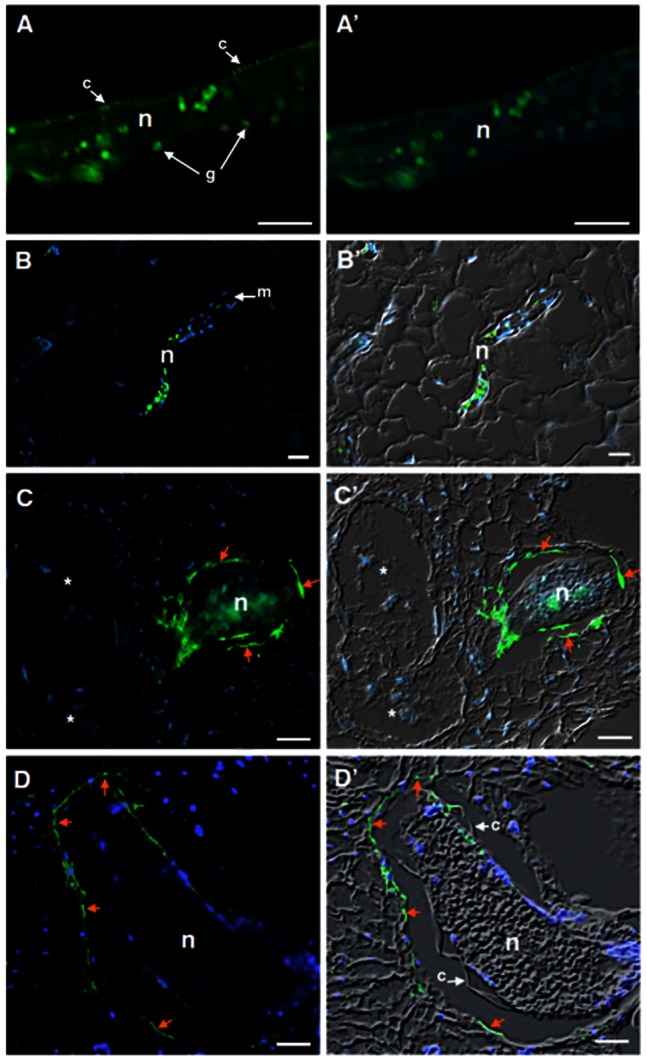
Immunodetection of FAR proteins in *Meloidogyne incognita* pre-parasitic J2 and during parasitism of *Arabidopsis thaliana* roots. (**A**) Cross nematode sections of pre-parasitic J2 displaying the protein localization at the nematode cuticle surface and circular granules structures within the posterior nematode body. (**B**) Localization of FAR proteins during nematode migration (**B-B′**), and nematode sedentary stages at 10 DAI (**C-C′**) and 21 DAI (**D-D′**) within the roots of *A. thaliana*. Arrows point out the accumulation of FAR along the nematode cuticle and adjacent cells surrounding the nematode body at 10 and 21 DAI. Micrographs on the left are overlays of Alexa-488 fluorescence (green) and DAPI-stained nuclei (blue). Micrographs on the right are overlays of an Alexa-488 fluorescence (green), DAPI-stained nuclei (blue) and differential interference contrast (grey). **c**, cuticle, **g**, granules, **n**, nematode, **m**, metacorpus, * giant cell. Bars = 10 µm.

To monitor the localization of *Meloidogyne* FAR on both pre-parasitic J2s and parasitic nematode life-stages within the host roots, the polyclonal antiserum raised against the full recombinant Gp-FAR-1 (Fig. S1) of the potato cyst nematode *G. pallida* was utilized [40]. Total extracted protein from *M. javanica* pre-parasitic J2s on protein gel blots confirmed the binding to this antiserum, while no binding was detected in the protein extracted from tomato roots (Fig. S1B). On sections of pre-parasitic J2s the antiserum cross-reacted with the cuticle surface of pre-parasitic J2s (Fig. 3A). Surprisingly, we could also frequently observe the binding of the antiserum to circular granules along the posterior part of the nematode body within the intestine (Fig. 3A). To analyze whether the FAR protein was secreted within the host roots during different parasitism stages, we performed an immunolocalization on infected material at 5, 7, 14 and 21 DAI (Fig. 3B–D). During migration within the root tissues, the FAR protein was often localized within the circular granules on the intestine of migratory juveniles (Fig. 3B). Although a large number of sections were screened containing migrating nematodes within the roots, we were unable to detect any secretion of FAR protein along the nematode migratory pathway. Nevertheless, as RKN become sedentary we could observe a strong fluorescence around the full nematode cuticle adjacent to the surrounding host cells, suggesting a continuous secretion of this protein into the intercellular space between the nematode body and the host cells at different developing sedentary stages (Fig. 3C–D). Control experiments using pre-immune serum showed no background on sections of pre-parasitic stages or infected roots at similar time points (Fig. S2).

### Expression of *mj-far-1* in tomato roots induces susceptibility to *M. javanica*

**Figure 4:**
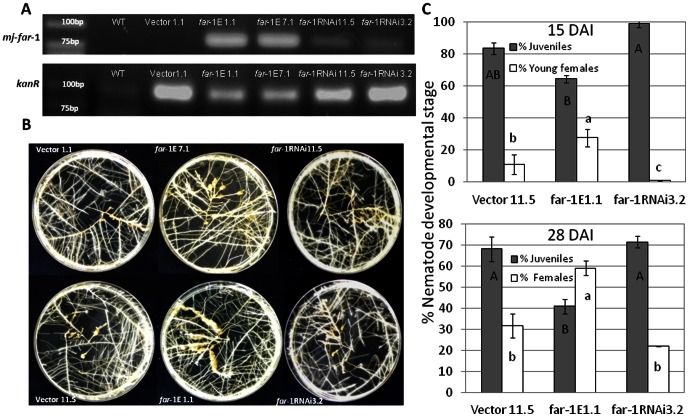
Constitutive expression of *mj-far*-1 in tomato hairy roots increases roots susceptibility to infection by the RKN *M.* *javanica*. **A.** RT-PCR confirmation of *mj-far-1* (upper gel) and *kanR* (lower gel) expression in tomato hairy roots lines *far*-1 E1.1, *far*-1 E 7.1, *far-1* RNAi3.2 and *far-1* RNAi11.5 compared with the respective control Vector 1.1 and WT 870. The expected size of the PCR product is 96 bp for the *mj-far*-1 and 81 for *kanR*. RT-PCR was performed on total RNA isolated from non infected transformed tomato hairy roots and WT 870 roots. **B.** Increased susceptibility of tomato hairy roots expressing *mj-far*-1 (*far-1*E1.1 and *far-1*E7.1) is accompanied by expanded galling production compared with *mj-far-1* dsRNA-expressing tomato hairy root lines (*far-1* RNAi3.2 and *far-1* RNAi11.5) and vector control (Vector 1.1. and Vector 11.5). **C.** *Meloidogyne* susceptibility/resistance of vector transformed roots and transgenic tomato roots expressing *mj-far-1*, or *mj-far-1* dsRNA-expressing lines, was measured as nematode developmental stages counted at 15 and 28 DAI. Roots were inoculated with 200 sterile pre-parasitic J2s and then assessed for juveniles, young females and mature females under the dissecting scope following staining with acid fuchsin dye. Note the significant (*P*≤0.05) increase in percentage of young females at 15DAI and increase in percentage of mature female at 28DAI in roots overexpressing *mj-far-1* in comparison with vector control roots. Data are expressed as means of 25 plants from each line; the experiment was repeated three times, giving consistent results. The percentage of each developmental stage is represented by a mean ± standard error. Different letters above the bars denote a significant difference (*P*≤0.05, analysis of variance) between tomato roots lines analyzed by Tukey-Kramer multiple comparison tests.

To gain a first insight into the effect of Mj-FAR-1 in host plants, *mj-far-1* was constitutively expressed in tomato hairy roots under the control of the cauliflower mosaic virus 35S promoter (CaMV35S) along with its signal peptide. Transgenic tomato hairy roots lines harboring a copy of *mj-far*-*1*, and control hairy root lines (with the empty vector PART27 [50]) were obtained from independent transformations, and confirmed through PCR (Fig. S3A). Overexpression *mj-far-1* root lines (hereafter called *far*-1 E1.1 and *far*-1 E7.1) and control lines (hereafter called Vector 1.1 and Vector 11.5) were subsequently used for southern blot analysis to confirm plasmid integration into the genome of the transformed roots (Fig. S3B). Additionally, the presence of expressed *mj-far*-*1* transcripts in transformed tomato roots were confirmed by RT-PCR, resulting in a fragment of 96 bp (Fig. 4A; upper panel). No expression of *mj-far*-*1* was detected in control root lines harboring an empty vector or in WT tomato roots (Fig. 4A; upper panel). The expression of the kanamycin resistance gene (*KanR*) gene in the different transformed root lines, was confirmed using *KanR* specific primers, resulting in the amplification of a 81 bp fragment, whereas no product amplification was observed for WT tomato roots (Fig. 4A; lower panel).

Phenotypic analysis of *mj-far*-*1* overexpressing root lines revealed no significant developmental alteration in root morphology or growth compared to control vector-transformed hairy roots (Fig. S4). To evaluate the effects of ectopic *mj-far-1* expression upon *M. javanica* inoculation, 200 freshly hatched pre-parasitic J2s were used for infection of each root system and followed during gall development (Fig. 4B). Infected roots of both overexpressing *far-1* E1.1 and *far-1* E7.1 lines were characterized by numerous and expanded galls compared with moderate and typical gall formation on control Vector 1.1 and Vector 11.5 lines (Fig. 4B), strongly suggesting that roots overexpressing *mj-far-1* were more susceptible to *M. javanica* infection compared to control lines. To further evaluate disease progress, both root lines overexpressing *mj-far*-*1* and control roots were dissected under a stereo-microscope for evaluation of nematode development at similar time points (Fig. 4C). Roots overexpressing *mj-far-1* (*far-1* E 1.1) were not only significantly (p<0.05) more susceptible to *M. javanica* infection, but also showed a fast nematode maturation compared to control lines (Fig. 4C). Hence, at 15 DAI we could observe a significant increase in the percentage of young developing females in overexpressing *mj-far-1* roots in comparison to the Vector 11.5 control roots (Fig. 4C). Likewise, at 28DAI a larger percentage of nematodes have developed into females in the *far-1* E1.1 line, whereas in control roots a higher percentage of nematodes were still under development (Fig. 4C). In addition egg masses number doubled in roots overexpressing *mj-far-1* compared with the control Vector 11.5 line (data not shown).

### Host-derived RNA interference (RNAi) of *mj-far-1* attenuate nematode development

Tomato hairy roots-derived RNAi was used to silence the expression of the *mj-far-1* gene within the nematode and to evaluate the consequential effects on parasitism (Fig. 4). The pHANNIBAL [51] vector containing the CaMV35S promoter was generated to drive expression of hairpin dsRNA complementary to *mj-far-1* within tomato hairy root (hereafter called *far*-1 RNAi3.2 and *far*-1 RNAi11.5). No significant root morphological differences were observed between the control and transgenic *mj-far-1*-RNAi tomato lines (Fig. S4). Infected tomato hairy root lines possessing the RNAi constructs demonstrate 80% reduction in *mj-far-1* transcript compared with vector control as measured by qRT-PCR conducted with RNA extracted at 15DAI (Fig. S3C). To evaluate disease progress on tomato *mj-far-1*-RNAi lines, both *far-1* RNAi3.2 and *far-1* RNAi11.5 were analyzed for their response upon *M. javanica* inoculation and gall development was followed (Fig. 4B). At 15 DAI the majority of nematodes extracted from the roots remained as second-stage juveniles, while only few nematodes were able to develop into young females in the *far-1* RNAi3.2 dsRNA expressing line when compared to both control or overexpression lines (Fig. 4C).

### Overexpression of *mj-far-1* positively affects giant cells and nematode development

**Figure 5:**
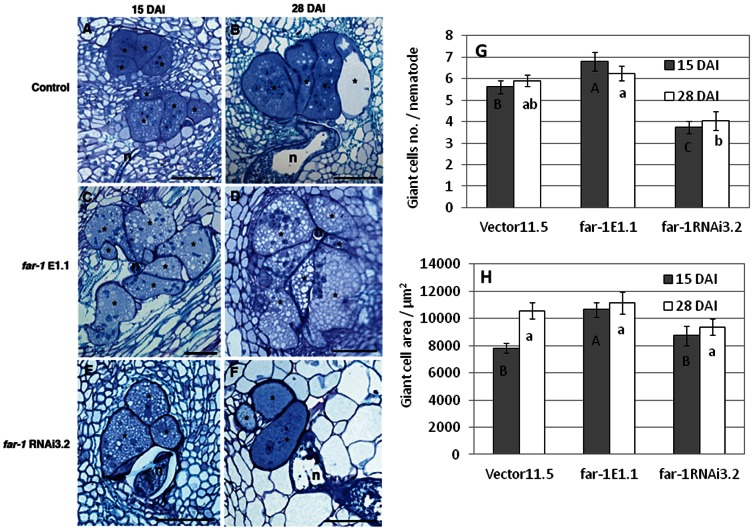
Longitudinal sections of *M.* *javanica* feeding sites in inoculated transgenic roots. Histological analysis of roots expressing *mj-far*-1 (**C, D**) and *mj-far-1*RNAi roots (**E, F**) compared with vector control (**A, B**) were conducted at 15 and 28 DAI. At 15DAI giant cell systems were more developed and consists of more giant cells in roots where an excess of Mj-FAR-1 (**G, H**). Sections were stained with toluidine blue. * giant cell; n, nematode; Bar = 100 µm. The average of giant cell number at 15 and 28 DAI of 60 gall cross sections for each tomato hairy root line is presented (G). Giant cell area was measured on 50 giant cells. Measurements are represented by a mean ± standard error. Statistically significant differences (*P*≤0.05, analysis of variance) were determined by Tukey-Kramer multiple comparison tests. Different letters above the bars denote a significant difference.

To evaluate whether the increase in root susceptibility observed was associated with any cellular alteration of the nematode feeding site, a detailed microscopic analysis was performed at different stages of gall development (15 and 28 DAI), for *far-*1 E1.1, *far-1* RNAi3.2 and Vector 11.5 lines (Fig. 5A–F). Surprisingly, the average number of giant cells in roots overexpressing *mj-far*-*1* (*far*-*1* E1.1) at 15DAI indicated a relatively higher number of giant cells associated with the respective developing nematodes compared to vector control roots (Figure 5G). Whereas for *far-1* RNAi3.2 roots at 15 DAI, a significant decrease in giant cells number associated with the respective developing nematode compared to vector control roots was observed (Fig. 5G). Additionally, the average area measured on giant cells of roots expressing *mj-far-1* (*far-1* E1.1) confirmed a significant increase size compared to both vector control roots or *far-1* RNAi3.2 at 15 DAI (Fig. 5H), indicating that giant cells of *mj-far-1* overexpressing line were larger (Fig. 5H). However, at 28 DAI the difference of giant cell development was less prominent among the three lines, suggesting that the process of gall formation is accelerated in the *far*-*1* E1.1 overexpressing line (Fig. 5H).

### Increased susceptibility of *mj-far-1* expressing roots is associated with suppression of jasmonic acid responsive genes

**Figure 6:**
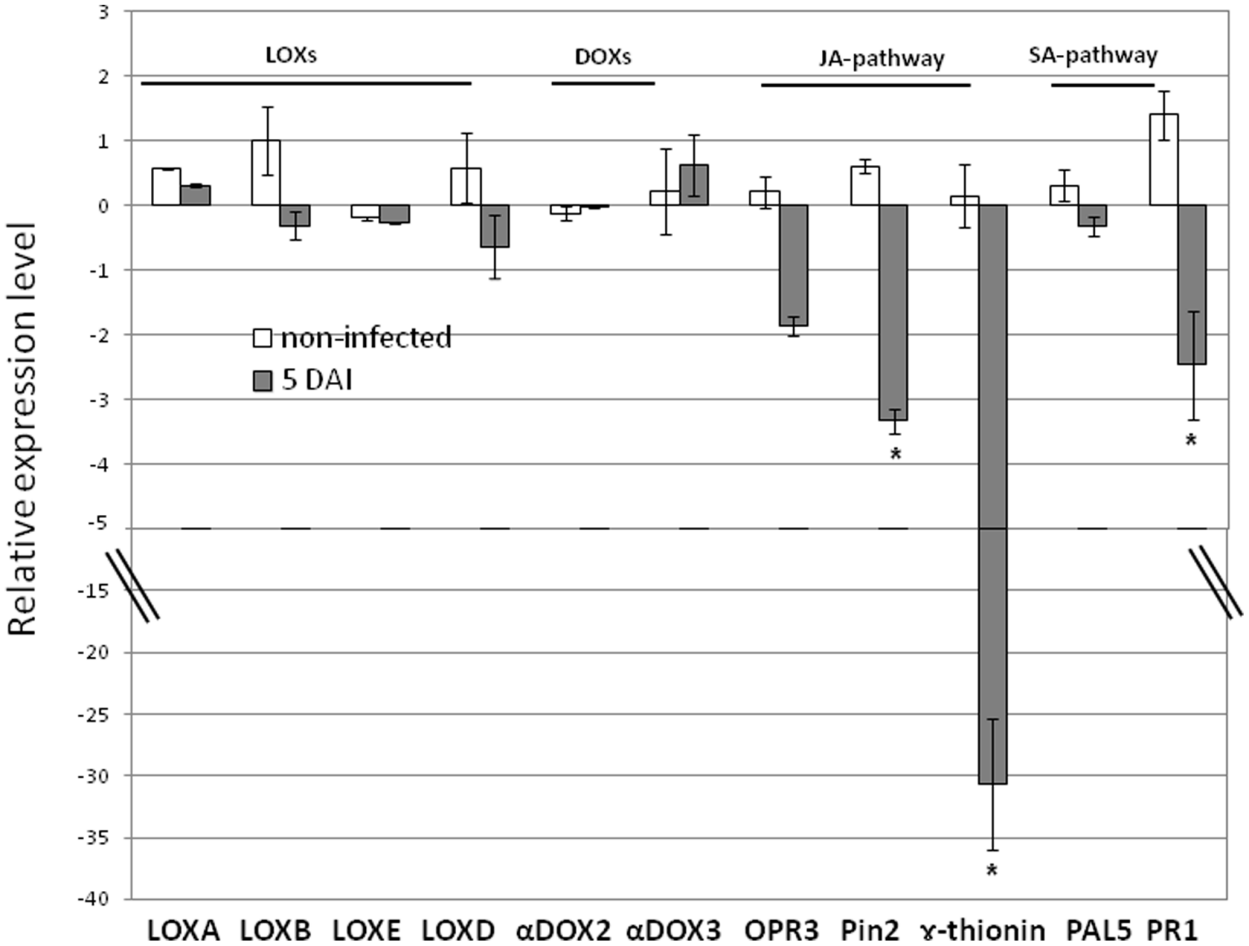
Analyzing Mj-FAR-1 functions in manipulating plant defense pathways. Expression level of 11 defence-related target genes in *mj-far-1* expressing root line *far-1* E1.1 compared with vector control prior and 5 DAI with *Meloidogyne javanica*. Total RNA was prepared from Vector 11.5 transformed control roots and roots expressing *mj-far-1* with/without infection. The graph shows the mean and standard error of the relative amount of transcripts of these genes in *mj-far-1* expressing roots (*far-1* E1.1) in comparison with vector transformed control roots (Vector 11.5) growing under the same conditions (vector control expression level set at zero). All target genes were normalized using the normalisation factor calculated as the geometric mean of the expression levels of three tomato endogenous reference genes *18S*, *actin* and *β-tubulin*. Each reaction was performed in triplicate and the results represented the mean of two independent biological replicates. Statistical significance of the differences between *far-1* E1.1 and Vector 11.5 transformed control roots were determined by Tukey-Kramer multiple comparison test, and significant differential expression (P≤0.05) is indicated with asterisks. The experiments were repeated three times with similar results.

To evaluate the contribution of LOXs and DOXs (hormones and oxylipin biosynthesis), the JA and the salicylic acid (SA) pathways (host defense mechanisms) toward the increased susceptibility of *mj-far-1* overexpressing roots, expression of a set of gene markers involved in these different pathways were investigated using qRT-PCR. The expression profile of oxylipin biosynthesis genes *TomLoxA* (U09026.1), *TomLoxB* (U09025.1), *TomLoxD* (U37840.1), *TomLoxE* (AY008278.1), *LEα-DOX2* (AY344540.1) and *LEα-DOX3* (AJ850958.1), indicated that expression of this group of genes show a similar pattern within both root Vector 11.5 and *far-1* E1.1 lines (Fig. 6). At 5 DAI, the expression analysis of *TomLoxD*, a 13-LOX encoding gene, was similarly induced upon nematode infection in both roots overexpressing *mj-far*-*1* or control vector transformed roots, indicating no effect of Mj-FAR-1 on the TomLoxD route (Fig. 6). Among the 9-LOXs genes, *TomLoxA*, *TonLOXB* and *TomLOXE* no significant effect was observed either by nematode infection or by *mj-far*-*1* overexpression (Fig. 6). Among the DOX encoding genes, while *LEα-DOX2* is highly suppressed upon nematode infection, its expression was not affected by excess of Mj-FAR-1 protein. On the contrary, the expression profile of *LEα-DOX3* was affected neither by nematode infection nor by *mj-far-1* overexpression (Fig. 6).

Transcription of pathogenesis-related (*PR-1*; accession no. M69247) and phenylalanine ammonia lyase (*PAL5*; accession no. M90692.1) genes whose induction patterns are often used as molecular markers for the activation of the SA signaling pathways were also analyzed. In addition, for the activation of JA signaling pathways, transcripts of proteinase inhibitor (*Pin2*; accession no. L21194), *OPR3* (12-oxophytodienoate reductase; accession no. A1486721) and *γ-thionin* (accession no. AJ133601.1) encoding genes were analyzed. Under non-infected conditions the transcript levels of the tested marker genes showed no significant down- or up-regulation in transgenic roots overexpressing *mj-far-1* having as reference the transcriptional levels observed for the control transformed hairy roots (Fig. 6). Among the JA responsive genes, both *Pin2* and γ*-thionin* genes displayed a significantly down-regulation in roots overexpressing *mj-far-1* compared with vector transformed roots at 5 DAI (Fig. 6; P<0.05). In the case of *OPR3* and the SA gene marker *PAL5* the observed changes were not significantly different and thus were not attributed to excess of FAR (Fig. 6). For all qRT-PCR analysis, transcripts were normalized against the geometric mean of the expression levels of three most stable tomato endogenous reference genes *18S*, *actin* and *β-tubulin*. Overall, our results suggest that overexpression of Mj-FAR-1 decreases transcript accumulation JA regulated.

## Discussion

During the last two decades the role of FAR of animal-parasitic nematodes have been extensively studied [34]–[36], however its biological role in their counterparts plant-parasitic nematodes was restricted to the cyst nematode *G. pallida* [40]. Although *in vitro* studies of *G. pallida* Gp-FAR-1 (Subfamily Heteroderinae) indicate its potential role on inhibiting the plant LOX [40], there is yet no information on the respective role of FAR proteins in root-knot nematodes species (Subfamily Meloidogyninae). Herein we examined the role of *Meloidogyne* FAR protein, by analyzing the transcription levels of *mj-far*-*1* during pre-parasitic stages (eggs and pre-parasitic J2), as well as parasitic stages in which the nematode is embedded within the host plant tissue. Transcriptional analysis along with immunocytochemical and functional studies of FAR during the sedentary stages suggest that FAR may play an active role during parasitism.

### Mj-FAR-1 share common features to other described FAR proteins

In the current study, we have identified one cDNA encoding *M. javanica* Mj-FAR-1, a member of the nematode-specific fatty-acid- and retinol-binding family of proteins. The Mj-FAR-1 protein shows similarity in its primary and secondary structures to previously described nematode FAR proteins (Fig. 1; [34], [36], [38]. A putative signal peptide, shared by other FAR proteins of other nematode parasitic species, was identified at the N-terminus of Mj-FAR-1, supporting the concept that this is a potentially nematode secreted protein (Fig. 1). Phylogenetic analysis of selected FAR proteins clearly distinguished and grouped the different nematode species in several clusters, in agreement with their trophic group (free-living *vs* parasitic nematodes) or even within each nematode parasitism strategy (sedentary *vs* migratory plant-parasitic nematodes, for example). These results might imply that despite the strong conservation in ligand binding characteristics previously suggested for this group of proteins [34]–[36], the extent in diversity of FAR proteins would enable or follow a particular mode of parasitism adaptation to certain hosts or nematode parasitism clades.

### Mj-FAR-1 is secreted into the inter-cellular space between the nematode body and host cells

Our immunocytochemical study of the Mj-FAR-1 on pre-parasitic J2 shows that FAR protein accumulates along the nematode cuticle surface. These results are in accordance to immunolocalization studies performed on pre-parasitic J2 of *G. pallida* Gp-FAR-1 [40]. Therefore, data on both root-knot and cyst nematodes suggest that this protein might be secreted by the hypodermis and interact at the level of the nematode cuticle surface. Our *in planta* immunocytological analyses during the early and late sedentary stages also showed the localization of FAR on the nematode surface. In addition we observe here a significant intercellular deposition of FAR protein at the extracellular space at the host-nematode interface during the sedentary parasitic stages (Fig. 3). All together, these findings place the FAR protein as parasitic component that might facilitate recruitment of cell mediators of immunity, presumably acting as part of the surface coat proteins that confer avoidance or manipulation of plant defense mechanisms. Similarly, the plant apoplasm has been considered as an important compartment where a number of proteins are delivered by root-knot nematodes [10]. Previously, subcellular localization of protease effectors secreted by phytopathogenic bacteria *Pseudomonas syringae* demonstrated their delivery into the plasma membrane, therefore promoting their biological function [52]. Apoplastic localization of effectors was also reported for phytopathogenic fungi, like *Cladosporium fulvum* during tomato infection [53]–[55] and by *Ustilago maydis* during maize infection [56]. Interestingly, several of these fungal and oomycete effectors have been shown to target many of the PR proteins which accumulate in the apoplast as part of the plant defense response [54], [56], [57]. Taking together, our findings suggest the idea that *Meloidogyne* FAR proteins secreted to the inter-cellular space sorrounding the nematode full body might interact with the host cells possibly facilitating nematode infection.

### The involvement of Mj-FAR-1 in manipulating plant defense pathways

Overexpression of nematode parasitism proteins in plant cells to study their *in planta* role have been described so far in several studies [58]–[65]. Herein, the function of Mj-FAR-1 during differential steps of parasitism was evaluated by the constitutive expression of the *mj-far*-*1* gene in tomato hairy roots, resulting in a substantial increase of the host susceptibility to *M. javanica* (Fig. 4). To better understand how these *mj-far*-*1* overexpressing roots became more susceptible to *M. javanica* infection, expression analysis of a set of genes belonging to the fatty acid metabolic pathways and that are involved in oxylipins biosynthesis e.g. JA, were analyzed. The effect of *mj-far*-*1* expression on plant root lipoxygenases (LOXs) and α-dioxygenases (α-DOXs) catalyzing the formation of fatty acid hydroperoxides was examined. Down-regulation of a component of the DOX pathway, the *LEα-DOX2*, was observed in both root lines *mj-far-1* overexpressing and vector control roots upon nematode infection. These findings illustrate that while *LEα-DOX2* is not precisely affected by FAR overexpression, oxylipins initiated by plant α-dioxygenases might exerts a specific function in regulating plant response to nematodes in addition to their critical role in tomato plant development [66]. Among the three 9-LOX isoforms, *TomLOXA*, *TomLOXB* and *TomLOXC* showed no obvious effect in their expression profile, either as function of Mj-FAR-1 or upon nematode infection. Unlike the 9-LOXs, the *TomLOXD* gene, acting upstream in JA biosynthesis pathway, showed a moderate up-regulation in both root lines *mj-far-1* overexpressing and vector control upon nematode infection, while no direct effect could be observed as a consequent result of FAR overexpression. These results show that DOXs as well as LOXs expression are not significantly affected by the ectopic Mj-FAR-1 expression upon *M. javanica* infection on tomato roots. In contrast to our results, Prior et al. (2001) showed inhibition of LOX by recombinant *G. pallida* rGp-FAR-1 protein. This differences might be explained by differences in substrate availability occurring in the *in vitro* system compared with the *in planta* system we used or it might be that FAR is involved in manipulating LOX activity but not its expression.

### Analyzing the interaction of FAR with JA pathway

Given that Jasmonic acid acts as signal activating the expression of various genes, such as the proteinase inhibitors, 12-oxophytodienoate reductase *(OPR3)* and γ-thionin [67]–[70] their expression profile was used to reflect the induction/suppression of the JA metabolic pathway. Both tomato proteinase inhibitor 2 (*Pin 2*) and *γ-thionin* were significantly down-regulated in roots overexpressing *mj-far-1*, while *OPR3* expression remained unstable during the independent experiments. Overall, these findings suggest that JA responsiveness pathways have been manipulated in roots overexpressing *mj-far*-*1* to support nematode parasitism. Interestingly, γ-thionins appear to play diverse roles as they present; i) anti-bacterial and/or anti-fungal activity [71], [72], ii) ability to inhibit mammalian cell growth by membrane permeabilization [73] and iii) the ability of inhibiting insect α-amylases and proteinases [74], [75]. Thus, these intrinsic characteristics described for plant γ-thionins, along with the significant suppression of γ-thionins as function of FAR, might contribute to the increased susceptibility response observed for roots overexpressing *mj-far*-*1*. Moreover, the observed suppression of proteinase inhibitor (*Pin2*) found in roots overexpressing *mj-far-1* might also facilitate nematode parasitism. Similarly, tomato mutant deficient in jasmonate synthesis, *def1*, fails to accumulate proteinase inhibitors in response to wounding and is considerably more susceptible than WT to attack by tobacco hornworm larva [76]. Furthermore, the implication of protease inhibitor in enhancing plant resistance to nematodes have been shown previously [77] and might further explain the increase in susceptibility observed in *mj-far-1* expressing roots. Following after, the SA-dependent systemic acquired resistance, through *PR1* and *PAL5* gene markers, indicate that expression of *PR1* merely, was consistently suppressed and thus might be involved in increased susceptibility observed in *mj-far-1* expressing roots.

Taking together, our data indicate that as a lipid binding protein, Mj-FAR-1, might regulate jasmonate-dependent defense responses to promote root susceptibility. These results are supported by the idea that JA is an important signaling molecule in plant defense [78]. Being an extracellular signal that is transmitted to intracellular targets [79], a modulation of JA activity as a result of FAR function might lead to qualitative and quantitative changes in cellular responses to extracellular signals. Moreover a number of studies clearly show that fatty acids per se are messenger and modulator molecules and are a key step in the signalling events that activate defence genes in tomato [80], [81] and thus might be directly or indirectly affected by FAR [82].

Yet our current understanding of the JA signaling in interactions with RKN and their plant hosts is not clear. Exogenous application of JA (as methyl jasmonate) in rice resulted in nematodes being less effective to counteract root-defense pathways, demonstrating a pivotal role of JA in rice defense against the RKN *Meloidogyne graminicola* [83]. Similarly, foliar treatment with methyl jasmonate significantly reduced the infection of RKN *M. incognita* in tomato plants [83]. Conversely, in maize, expression studies together with genetic approaches provided compelling evidence of JA as a negative regulator of plant resistance: a mutant lacking ZmLOX3 led to increased levels of JA and higher susceptibility to the RKN *M. incognita* [84]. Similar to maize, the use of tomato JA mutant lines demonstrated that intact JA signaling is required for tomato susceptibility to the RKN *M. javanica*, although is not involved in *Mi*-genes-mediated resistance [26]. Our results suggest that the susceptibility of *mj-far*-*1* overexpressing plants is likely associated with suppression of JA pathway at the earlier parasitic stages as indicated by down-regulation of two major JA responsive genes *Pin2* and *γ-thionin*.

To conclude, the study of Mj-FAR-1 protein during *M. javanica*/tomato interaction provided novel information on the putative role of FAR proteins during nematode infection. The fast maturation of giant cells and the accelerated nematode development caused by *mj-far*-*1* overexpression suggest the involvement of FAR in gall development. Taking all data together, we suggest that the FAR protein may have a key role in manipulating plant defense signaling through regulating the availability of lipid signals to promote successful plant parasitism.

## Materials and Methods

### Plant materials and growth conditions

Tomato (*Solanum lycopersicon*) cv. Avigail )870) was used as the background line for transformation. For germination, tomato seeds were soaked in sterile water for 1 h at 32°C, treated with 1.4% (v/v) NaOCl for 10 min, washed several times with sterile water for 5 min, and then plated on standard strength Gambourg's B5 salts medium (Duchefa, Haarlem, The Netherlands), supplemented with 2% sucrose and solidified with 0.8% Gelrite agar (Duchefa). Seeds were kept in a growth chamber at 26°C, with a 16/8-h photoperiod at 120 µmol m^−2^ s^−1^. One week after germination, tomato roots sections were subculture, by placing one root per Petri dish (Miniplast, Ein Shemer, Israel), containing B5 medium. Petri dishes were kept horizontally for further root growing and branching, for an additional week at 26°C under dark conditions prior to nematode inoculation.

### Nematode culture and infection assays

*Meloidogyne javanica* was propagated on greenhouse-grown tomato *Solanum lycopersicon* cv. Avigail (870). Nematode egg masses were extracted from roots with 0.05% (v/v) sodium hypochlorite (NaOCl) following by sucrose floatation [85]. For sterilization, eggs were placed on a sterile Whatman® filter holder (Whatman International Ltd, Dassel, Germany) with a cellulose acetate filter membrane (Sartorius Stedim Biotech GmbH, Goettingen, Germany, pore size 5 µm). Eggs on the filter were exposed for 10 min to 0.01% (w/v) mercuric chloride (HgCl_2_) (Sigma-Aldrich, St Louis, MO), followed by 0.7% Streptomycin solution (Sigma-Aldrich), and three washing steps of 50 ml sterilized distilled water [86]. Sterile eggs were then collected from the membrane and placed on 25-µm opening sieves in 0.01 M MES (Sigma-Aldrich) buffer under aseptic dark conditions for 3 days. Freshly hatched pre-parasitic J2s, were then collected in a 50 ml falcon tube. For nematode infection tests, wild-type (WT) tomato roots and transgenic lines, growing on standard strength Gambourg's B5 salt medium (Duchefa), were inoculated with 200 sterile freshly hatched *M. javanica* pre-parasitic J2s. Plates were left uncovered in a laminar flow hood until water had completely soaked into the medium [87]. The inoculated and non-inoculated roots were left to grow horizontally under darkness and root samples were taken either for RNA extraction or assessment of nematode development at designated time points after inoculation.

### Isolation of *mj-far-1* DNA and cDNA full length

Genomic DNA was isolated from *M. javanica* eggs and pre-parasitic J2s using the CTAB (cetyltrimethylammonium bromide) plant DNA extraction method according to Goetz et al. (2001) [88]. The Universal Genome Walker kit (Clontech) was used to determine both the 5′ and 3′ flanking sequences, of *M. javanica far-1* gene using an adaptor primer (AP1) supplied by the manufacturer along with a reverse *far-1* specific primer, GSP2-rev (GTGTAAAGCTTTTCGCTCTTTTCCTTC), designed specifically for amplifying the 5 ′ flanking region. To amplify the 3′ flanking region an adaptor primer (AP2) along with a forward *far-1* specific primer GSP2-for (GCAAAGGAATCTCTAAAGACCAACTTCC), were used. Subsequently, all purified PCR products were cloned into pGEM-T (Invitrogen, Carlsbad, CA, USA) easy vector for sequencing. The derived sequences data were used to design the nested primers GSP2-nes-for (CCAGCCTAAATGAGAGAAAATGCTTTG) and GSP2-nes-rev (GGCGACTTCTTTCAAGATTTTCTTGTC) used to amplify the full length DNA *mj-far*-*1*, which was deposited in GenBank under the accession no. JX863901. To get the *mj-far*-*1* full-length cDNA, freshly hatched *M. javanica* pre-parasitic J2 were first homogenized using glass beads and liquid nitrogen following total RNAs extraction using an InviTrap Spin Plant RNA Mini Kit (Invitek, Robert-Rössle-Str, Berlin. Germany). To remove contaminating genomic DNA, RNA samples were incubated in presence of 10 units of TURBO DNA-free™ DNASE (Applied Biosystems, Foster City, CA). DNA-free RNA was converted into first-strand cDNA using the Verso™ cDNA Synthesis Kit (ABgene, Epsom, UK). *mj-far-1* cDNA amplification product with the primer pair longFAR-for (CATTCAATCAACAAGCCTACTTACTCTC) and longFAR-rev (TCTTGCTATTGCGAATCAAAGCATTTTC) was subsequently cloned into pGEMT-far vector for sequencing confirmation.

### Sequence comparisons and secondary structure predictions

Sequence analysis was conducted through programs available at the ExPASY molecular biology server (http://expasy.org/proteomics). The signalP 3.0 [89], the PSORT II [90] along with the TargetP [45] algorithms were used to predict the location of the signal peptide and its cleavage site. Calculation of the predicted Mj-FAR-1 molecular weight and isoelectric point were performed through the ProtParam program [91]. Secondary structure prediction of the protein sequence was performed using the PHD [47] and Jpred algorithms [48]. FARs sequence of other nematodes species were obtained by performing BLAST searches against a range of databases available online, such as www.nematode.net, www.nematodes.org and www.ncbi.nlm.nih.gov. The ClustalW program [92] was used to generate an alignment of the FARs protein sequences from *Caenorhabditis elegans*, animal- and plant-parasitic nematodes. Phylogenetic analysis were conducted on the alignment using the maximum likelihood protein sequence parsimony method [93] and the unweighted pair group method with arithmetic mean [94]. Trees were rooted against the FAR protein sequence of the cyst nematode, *Globodera pallida* Gp-FAR-1. All methods produced trees of the same topology, and a typical resulting tree with bootstrap values is presented.

### RNA isolation and qPCR

Gene transcripts were quantified by real-time RT-PCR on total RNA extracted from eggs, freshly hatched pre-parasitic J2s, and parasitic stages of *M. javanica* within infested tomato roots at four different time-points: 6 and 48 h, and then at 5, 15 and 28 days after inoculation (DAI). Ten root systems were pooled for each time point corresponding to different life cycle stages within the roots while non-infected roots treatment was used as control. Prior to all RNA extractions, samples were mechanically disrupted and homogenized by using glass beads and liquid nitrogen. RNA then was extracted from the homogenized samples using the InviTrap Spin Plant RNA Mini Kit (Invitek, Robert-Rössle-Str, Berlin, Germany). To remove contaminating genomic DNA, RNA samples were incubated in the presence of 10 units of TURBO DNA-free™ DNASE (Applied Biosystems, Foster City, CA). DNA-free RNA was converted into first-strand cDNA using the Verso™ cDNA Synthesis Kit (ABgene, Epsom, UK) and qRT-PCR reactions were performed using the SYBR-Green ROX Mix (ABgene). qRT-PCR primers were designed using the Primer Express software (Applied Biosystems, Tables S1 and S2). The subsequent real-time PCR reaction contained 3.4 µl of the cDNA in a total volume of 10 µl, consisting of 1× SYBR-Green ROX Mix (ABgene) and 150 nM forward primer and 150 nM reverse primer subjected in real-time PCR plasticware (Axygen, Union City, CA). All PCR cycles began with 2 min at 50°C then 10 min at 95°C, followed by 40 two-step cycles comprising 10 s at 95°C and 1 min at 60°C. After PCR reaction, a melting curve was generated by gradually increasing the temperature to 95°C to test for amplicon specificity. For qPCR a mixture of all cDNAs was used for all treatments, as a template for calibration curves designed for each pair of primers. Each reaction was performed in triplicate and the results represented the mean of two independent biological experiments. Two constitutively expressed genes the small ribosomal RNA (*18S*; GenBank accession no. AF442193.1) and elongation factor-1α genes (*EF-1α*; GenBank accession no. U94493.1) were the endogenous controls for the *M. javanica* gene expression analysis (Table S1). Three constitutively expressed genes the actin (*ACT*; GenBank accession no. U60482.1), *β-tubulin* (*TUB*; GenBank accession no. NM_001247878.1) and *18S* gene (GenBank accession no. BH012957.1) were the endogenous controls for tomato gene expression analysis (Table S1). Transcript levels were normalized for each sample with the geometric mean of the corresponding selected housekeeping genes. All of the housekeeping genes were confirmed to display minimal variation across the treatment and were most stable housekeeping genes from a set of tested genes in a given cDNA sample [95].

Values were expressed as the increase or decrease levels relative to a calibration sample. The control reactions were included as follow: PCR negative control without cDNA template to confirm that there were no nonspecific PCR products (NTC), and a second reaction contained mRNA that had not been subjected to a reverse transcriptase reaction (NRT control). Statistical differences between treatments and/or roots lines were calculated by LSD, according to Tukey-Kramer multiple comparison test at *P*≤0.05. For confirmation of all qRT-PCR results, expression of the subset of genes was analyzed in another two independent experiments, producing the same results.

### Immunolocalization of Mj-FAR-1 during pre-parasitic and parasitic stages

Serum directed against the recombinant rGp-FAR-1 from *G. pallida* was kindly provided by Prof. John Jones (James Hutton Institute, Scotland). The antiserum was tested by Western blotting of proteins extracted from *M. javanica* pre-parasitic J2s and tomato roots as negative control with methods described previously by Duncan et al. (1997). The immunolocalization assay on pre-parasitic juveniles was performed essentiality as described by Vieira et al. (2011). Freshly hatch pre-parasitic J2s were collected to a 2 ml Eppendorf tubes, centrifuged for 5 min at 3,000 rpm, and immediately resuspended in fixative (4% formaldehyde in 50 mM Pipes buffer, pH 6,9), refreshed twice and kept at 4°C for 7 days. Samples were then prepared for immunocytochemical analysis according to Vieira et al. (2011). Slide containing sections of pre-parasitic J2s were incubated with 1∶50 primary anti-Gp-FAR-1 serum and 1∶200 secondary Alexa 488 goat anti-rabbit IgG antibodies (Molecular Probes). As control nematodes sections were incubated with pre-immune serum in the absence of primary antibody. Representative cytological images of pre-parasitic J2s for protein localization were collected using a digital camera (AxioCam, Zeiss). For localization of FAR proteins in nematode plant infected roots, roots of *Arabidopsis thaliana* cv. Columbia grown *in vitro* were inoculated with the surface-sterilized *M. incognita* pre-parasitic J2s population Calissane. Dissected roots and galls, taken at 5, 7, 14 and 21DAI, were fixed with 4% formaldehyde in 50 mM Pippes buffer (pH 6.9). Samples were then prepared for immunocytochemical analysis according to Vieira et al. (2011). Gall sections were incubated with 1∶50 primary anti-Gp-FAR-1 serum and 1∶300 secondary Alexa 488 goat anti-rabbit IgG antibodies (Molecular Probes). As control gall sections were incubated with pre-immune serum in the absence of primary antibody. Slides were mounted with ProLong antifade medium (ProLong antifade kit; Invitrogen Molecular Probes), and observed with a microscope (Axioplan 2, Zeiss) equipped for epifluorescence and differential interference contrast optics. Representative cytological images of migratory and parasitic stages of nematodes within the plant root for protein localization were collected using a digital camera (AxioCam, Zeiss). Images of immunolabelled sections, differential interference contrast transmission, and DAPI-stained DNA (nuclei) were overlaid.

### Plasmid construction and generation of transgenic tomato roots

All PCR amplification used for plasmids construction were performed using the Expand High Fidelity plus PCR System (Roche) according to the manufacturer's instructions. For the *mj-far*-*1* overexpression construct, the coding region of *mj-far-1* was amplified from the full-length cDNA pGEMT-far plasmid using gene-specific primers designed to create the *EcoR*I and *Cla*I restriction sites in the forward FAR1.OE.for (CTCTCTTAATAAAGAATTCAAAATGAGCCG) and reverse FAR1.OE.rev (CAAAGCATTATCGATCATTTAGGCTGGTGC) primers, respectively. The full length *M. javanica* cDNA (612 bp) was then cloned into the pHANNIBAL vector [51] at the *EcoR*I and *Cla*I restriction sites. The 2968 bp cassette containing the cauliflower mosaic virus 35S promoter with the *mj-far-1* coding sequence and the nopaline synthase (OCS) terminator were then isolated by restriction digestion with *Not*I and subsequently cloned into the pART27 binary vector [50]. The identity, orientation, and junctions of the resulting pART_OE construct were confirmed by PCR and sequencing. Both empty vector control pART27 and the constructed pART27_OE plasmids were subsequently used for *Agrobacterium rhizogenes* mediated root transformation as described below. For host-derived RNAi vector construction a 570 bp PCR product of *mj-far-1* was amplified using the gene-specific primers designed to create the *ClaI-KpnI* and *XbaI-EcoRI* restriction sites in the forward FAR1.RNAi.for (AATAATCGATTTTGCCGGTACCTTGGC) and the reverse FAR1.RNAi.rev (CTCATCTAGACTGGTGCAGCAGCGAATTCTGGT) primers, respectively. The PCR amplicon was then cloned in the sense and antisense orientation at the *ClaI-KpnI* and *XbaI*-*EcoRI* restriction sites, respectively, of the pHANNIBAL (RNAi) vector containing the CaMV35S promoter (Wesley et al., 2001) to express hairpin dsRNA of *mj-far-1* in transformed tomato hairy roots. The subsequent pHANNIBAL vector was subcloned as *NotI* fragments into the binary vector pART27 [50] and the construct was transformed into tomato roots via *Agrobacterium rhizogenes*.

### *Agrobacterium rhizogenes* *-*mediated root transformation-production of hairy root cultures

The binary vector pART27_OE and the empty vector control pART27 were transferred into *Agrobacterium rhizogenes* ATCC 15834 by electrotransformation [96]. Individual cotyledons were excised from 8- to 10-day-old tomato seedlings and immersed in an *A. rhizogenes* suspension (OD_600_ = 1.0) for 15 min. The excised cotyledons were then placed on a standard strength Gambourg's B5 salts medium for 3 days of co-cultivation, following by transferring to B5 agar media supplemented with the antibiotics Kanamycin (50 µg/ml) (Duchefa Biochemie) and the Timentin (300 µg/ml) (Ticarcillin Disodium/Potassium Clavulanate 15∶1) (Duchefa Biochemie). Within 7 to 10 days of incubation at 25°C under the light, roots emerged on the surface of the cotyledons. Hairy roots were transferred to Gamborg's B5 medium (Sigma) containing 0.8% gelrite and Kanamycin (50 µg/ml).

### Transgene integration in hairy roots

The presence and expression of transgenes in the tomato hairy roots genome were confirmed by genomic PCR, Southern blot, and RT-PCR. PCR amplification confirmed the presence of KanR gene by amplification of PCR product of 662 bp with the primer set KanR genomics (Fig. S3A, see Supporting Information). Southern blotting to confirm T-DNA integration into the tomato roots genome was performed using DIG-labeled probes according to the manufacturer's protocols (Roche Molecular Biochemicals, Mannheim, Germany) (Fig. S3B, see Supporting Information). Genomic DNA of transformed roots was digested with EcoRI, which does not cut within the T-DNA, and hybridized with a labelled 380 bp *KanR* amplicon using the primer set KanR-F (CCGGTTCTTTTTGTCAAGAC) and KanR-R (AGAAGAACTCGTCAAGAAGG). No hybridization signal was observed in non-transformed plants. In contrast, the presence of *KanR* fragment was detected in transgenic lines, showing that T-DNA was integrated into transgenic root genome (Fig. S3B, see Supporting Information). RT-PCR using RNA extracted from hairy roots and non-transformed tissues were used to confirm that the transgenes were transcribed *in planta*. To quantify the expression of *mj-far-1* in *far-1* RNAi3.2 and vector control roots, total RNA was extracted from homogenized galls samples of both lines collected at 15 DAI using the InviTrap Spin Plant RNA Mini Kit (Invitek, Robert-Rössle-Str, Berlin, Germany). qRT-PCR was performed using the primer sets designed for *mj-far-1* as shown in Table S2, and transcript levels were normalized for each sample with the geometric mean of expression of two *M. javanica* selected housekeeping genes, the *18S* and *EF1α* (Fig. S3C). Three independent qRT-PCR reactions were conducted with similar results.

### Morphological analysis of infected root mutants

For morphological analysis, nematode-infected transformed tomato roots were fixed in 0.25% glutaraldehyde, 4% paraformaldehyde in 50 mM PBS pH 7.2, and then dehydrated and embedded in Technovit 7100 (Heraeus Kulzer) as described by the manufacturer. Embedded tissues were sectioned (3 µm) and stained in 0.05% toluidine blue and posteriorly mounted in Depex (Sigma-Aldrich). Microscopy was performed using bright-field optics and images were collected with a digital camera (Axiocam; Zeiss).

### Evaluation of tomato roots response to RKNs

To monitor nematode development in WT and transgenic tomato root lines, root systems grown in monoxenic culture were harvested at 15 and 28 DAI. Infected roots were stained with acid fuchsin solution (Sigma-Aldrich) (17.5 mg acid fuchsin, 500 ml ethanol and 500 ml acetic acid) for O.N. Stained roots were washed three times in distilled water and stored as described previously [97]. Distained roots were then mounted in tap water and galls were dissected under a stereo-microscope (Olympus SZX12). For analysis of nematode development, the number of J2, J3/4, young females and mature females were counted at 15 and 28 DAI. Mean values of sedentary J2, J3/J4 juveniles and females were obtained by screening 25 replicates per each line. Infection tests were repeated three times with similar results, results of only one experiment is presented. Statistical differences were determined for each independent experiment by all mean comparison, using the Tukey-Kramer test with an alpha level of 0.05 using JMP software (SAS Institute, Cary, NC).

## Supporting Information

Table S1 Forward and reverse primers sequences used for PCR, RT-PCR for transgenic roots confirmation and probe amplification, along with primers sets for internal control genes used to normalize gene expression throughout the qRT-PCR expression analysis.
(DOCX)

Table S2 Overview of the target genes used throughout this study, primer pair used for qRT-PCR, the defense pathway in which each gene is mainly involved along its accession no. and reference are shown.
(DOCX)

Figure S1 **Mj-FAR-1 characterization.** **A.** Amino acid sequence alignments of the FAR proteins of *Globodera pallida* (Gp-FAR-1) and *Meloidogyne javanica* (Mj-FAR-1), GeneBank accession number: CAA70477.2 and JX863901 respectively. Sequences were aligned using ClustalW and prepared for display by BOXSHADE. Identical amino acids are shaded in black, and similar substitution in gray. **B.** Identification of Mj-FAR-1 in infective J2s of *M. javanica*. Western blot analysis of proteins extracted from *M. javanica* J2s and tomato roots as control probed with an antiserum raised against rGp-FAR-1. A single band of molecular mass approx. 20 kDa is detected. Lane M, molecular mass standard proteins (PageRuler™; Thermo scientific Pierce), whose molecular masses are as indicated (in kDa) at the left. Lane R; protein extracted from tomato roots (R); Lane J2; Protein extracted from *M. javanica* infective J2s.
(TIF)

Figure S2 **Control images of Mj-FAR-1 immunodetction.** For the control pre-immuno serum was used in place of the primary antibody, in sections of pre-parasitic J2s (**A**), and in roots of *Arabidopsis thaliana* infected with *M. incognita* at 7 (**B**) and 21 (**C**) DAI. Left panel, fluorescence images of Alexa-488 (green), and DAPI-stained nuclei (blue) appear paired with corresponding DIC overlays on the right panel. n, nematode; * giant cell. Bars = 10 µm.
(TIF)

Figure S3 **Detection of transgenic hairy roots by PCR and Southern hybridization.** **A.** PCR amplification of *kanR* in tomato hairy roots lines transformed with pART27_OE (*far*-1 E1.1 and *far*-1E 7.1) as well as vector control roots transformed with pART27 (Vector 1.1. and Vector 11.5) along with the respective WT tomato roots 870. Positive lines resulted in DNA amplification of PCR product of 660 bp while no product was amplified in WT roots. **B.** To determine the integration of pART27 or pART27_OE plasmids into the tomato genome Southern blot analysis was conducted by digesting the genomic DNA of vector transformed lines (Vector 1.1. and Vector 11.5), roots expressing mj-far-1 (*far*-1 E1.1 and *far*-1 E 7.1) and WT roots (870) with *EcoR*I which does not cut within the *KanR* resistance gene. The panel shows results obtained from using *kanR* as a specific probe. **C.** Host-derived RNA-interference (RNAi) of *mj-far-1*. RNA was extracted from tomato lines possessing the RNAi construct and vector control roots at 15DAI and *mj-far-1* expression was analyzed by qRT-PCR. *mj-far-1* transcripts were normalized using the geometric mean of the expression levels of two most stable nematode endogenous reference genes *18S* and EF-1α. Each reaction was performed in triplicate and the results represent the mean ± standard error of two independent biological replicates.
(TIF)

Figure S4 **Phenotypic observation of tomato roots constitutive expressing** ***mj-far*** **-1,** ***mj-far-1*** **dsRNA expressing lines and vector transformed roots.** Expression of *mj-far*-1 did not result in alteration in root morphology as indicated by root growth rate and branching. Roots of *far-1* E1.1, *far-1* E7.1, *far-1*RNAi11.5, *far-1*RNAi3.2, Vector 11.5 and Vector 1.1 were cultured on B5 medium for 5 days then roots length and branching were documented.
(TIF)
