# Supplementary material for: Fatty Acid-and Retinol-Binding Protein, Mj-FAR-1 Induces Tomato Host Susceptibility to Root-Knot Nematodes
Source: PLoS One. 2013 May 22;8(5):e64586. doi: 10.1371/journal.pone.0064586 (PMC3661543; doi:10.1371/journal.pone.0064586)
Supplement: Table S1 — Forward and reverse primers sequences used for PCR, RT-PCR for transgenic roots confirmation and probe amplification, along with primers sets for internal control genes used to normalize gene expression throughout the qRT-PCR expression analysis. (DOCX) [file pone.0064586.s001.docx]

**Table S1.** Forward and reverse primers sequences used for PCR, RT-PCR for transgenic roots confirmation and probe amplification, along with primers sets for internal control genes used to normalize gene expression throughout the qRT-PCR expression analysis.

| ***Gene*** | ***Accession no.*** | ***Forward 5'-3'*** | ***Reverse 5'-3'*** |
| --- | --- | --- | --- |
| ***KanR* (genomics PCR)** | CAC86252.1 | CCGGTTCTTTTTGTCAAGAC | AGAAGAACTCGTCAAGAAGG |
| ***KanR* (RT-PCR)** | CAC86252.1 | AGTACGTGCTCGCTCGATGC | ATGGCTGATGCAATGCGG |
| **18s (*M. javanica*)** | AF442193.1 | GCCTGCGGCTTAATTTGACT | CACCACCATCCACTGAATCATG |
| **EF-1α (*M. javanica*)** | U94493.1 | TGGTGGTATCGACAAGAGAACG | CAACTTGTCCAACACCCAAGC |
| **18s (Tomato)** | BH012957.1 | GAAACGGCTACCACATCCAAG | CCCCGTGTTAGGATTGGGT |
| **Actin Tom52 (Tomato)** | U60482.1 | ATGTATGTTGCCATCCAGGCT | TGTGGCTGACACGATCTCCA |
| **β-Tubulin (Tomato)** | NM_001247878.1 | ACCATTTGATCTCTGCAACCATG | TTCACAGCCAATTTCCTCAGG |
