# Supplementary material for: Fatty Acid-and Retinol-Binding Protein, Mj-FAR-1 Induces Tomato Host Susceptibility to Root-Knot Nematodes
Source: PLoS One. 2013 May 22;8(5):e64586. doi: 10.1371/journal.pone.0064586 (PMC3661543; doi:10.1371/journal.pone.0064586)
Supplement: Table S2 — Overview of the target genes used throughout this study, primer pair used for qRT-PCR, the defense pathway in which each gene is mainly involved along its accession no. and reference are shown. (DOCX) [file pone.0064586.s002.docx]

**Table S2.** Overview of the target genes used throughout this study, primer pair used for qRT-PCR, the defense pathway in which each gene is mainly involved along its accession no. and reference are shown.

| ***Gene*** | ***Accession no.*** | ***Forward 5'-3'*** | ***Reverse 5'-3'*** | ***Pathway involved*** | ***Source*** |
| --- | --- | --- | --- | --- | --- |
| ***mj-far*-1** | JX863901 | CGAGAAGCACAGCGAATATGC | GCGGAGCTCGTTTGCCT | Lipid signalling | this study |
| **TomloxA** | U09026.1 | AGAACTCCTTCGAACAGACGGT | TCCTCCACGCGGTTTTACTATC | Oxylipin biosynthesis | this study |
| **TomloxB** | U09025.1 | CCTATGCCTCAAGAACTCTGCTC | CTCCGTCTGGATGTGGCAA | Oxylipin biosynthesis | this study |
| **TomloxE** | AY008278.1 | TTGGGTGAGATCGAGGATCG | CACTGGTGGGAAAGAGGAAAGT | Oxylipin biosynthesis | this study |
| **TomloxD** | U37840.1 | TCACCAACTCGTCAACCACTG | ATGCATCGCGCTTAACTGC | Oxylipin biosynthesis | this study |
| **LEαDOX2** | AY344540.1 | TTGCAGCAATGCGAACCA | CCAAAATTGCACCTCCAACG | Oxylipin biosynthesis | this study |
| **LEαDOX3** | AJ850958.1 | GTAACAGTGGCCTCCAAGCTCT | TAACCACGCGCACGCTATC | Oxylipin biosynthesis | this study |
| **PIN2** | L21194 | TTGCTCTCCTCCTTTTATTTGG | GCAAGCCTTGGCATGTTC | JA response | [69] |
| **OPR3** | A1486721 | TTGGCTTAGCAGTTGTTGAAAG | TACGTATCGTGGCTGTGTATCA | JA biosynthesis | [70] |
| **γ-thionine** | AJ133601.1 | CAATGCTTGTCATGGCTACTGG | CTCACATACCGAGGCACAATTC | JA response | this study |
| **PR-1** | M69247 | GAGGGCAGCCGTGCAA | CACATTTTTCCACCAACACATTG | SA response | [25] |
| **PAL5** | M90692.1 | CAATGGCTTCTTACTGCTCGG | CATCTTGGTTGTGTTGCTCAGC | SA response | [70] |
