# Supplementary figures and images for: Fatty Acid-and Retinol-Binding Protein, Mj-FAR-1 Induces Tomato Host Susceptibility to Root-Knot Nematodes
Source: PLoS One. 2013 May 22;8(5):e64586. doi: 10.1371/journal.pone.0064586 (PMC3661543; doi:10.1371/journal.pone.0064586)

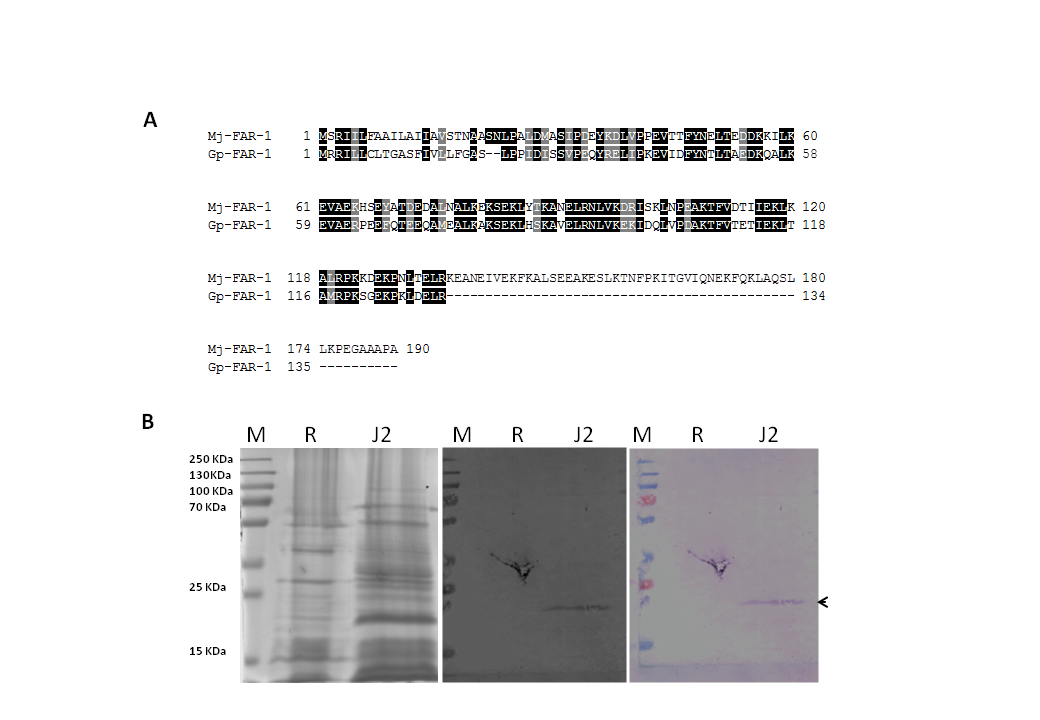

Supplement: Figure S1 — Mj-FAR-1 characterization. A. Amino acid sequence alignments of the FAR proteins of Globodera pallida (Gp-FAR-1) and Meloidogyne javanica (Mj-FAR-1), GeneBank accession number: CAA70477.2 and JX863901 respectively. Sequences were aligned using ClustalW and prepared for display by BOXSHADE. Identical amino acids are shaded in black, and similar substitution in gray. B. Identification of Mj-FAR-1 in infective J2s of M. javanica. Western blot analysis of proteins extracted from M. javanica J2s and tomato roots as control probed with an antiserum raised against rGp-FAR-1. A single band of molecular mass approx. 20 kDa is detected. Lane M, molecular mass standard proteins (PageRuler™; Thermo scientific Pierce), whose molecular masses are as indicated (in kDa) at the left. Lane R; protein extracted from tomato roots (R); Lane J2; Protein extracted from M. javanica infective J2s. (TIF) [file pone.0064586.s003.tif]

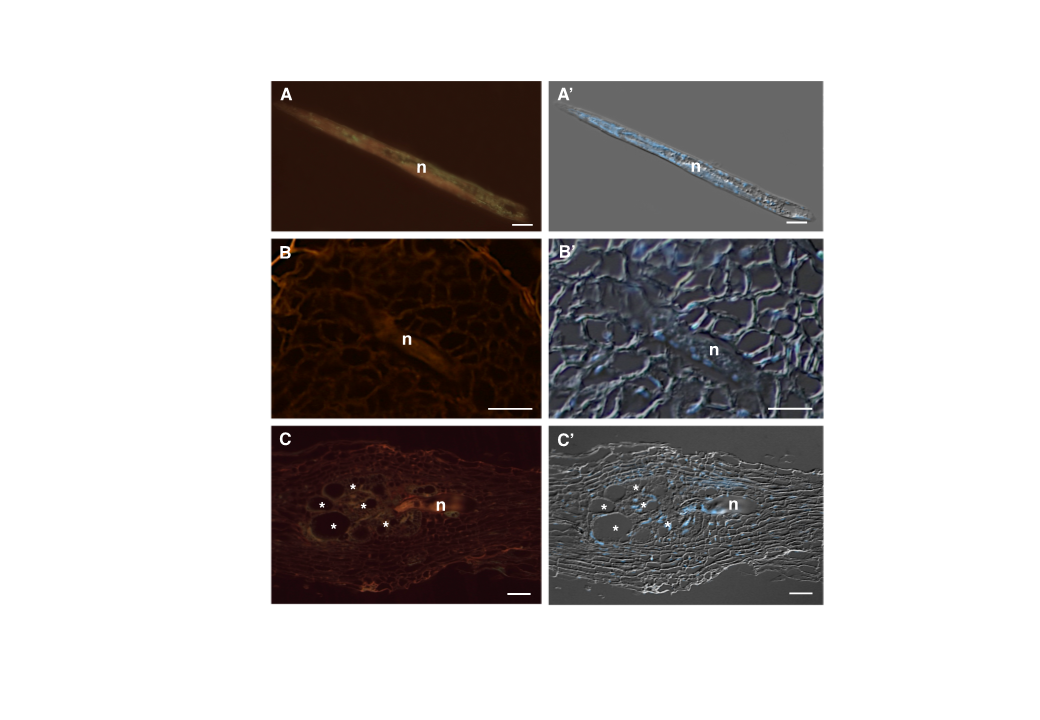

Supplement: Figure S2 — Control images of Mj-FAR-1 immunodetction. For the control pre-immuno serum was used in place of the primary antibody, in sections of pre-parasitic J2s (A), and in roots of Arabidopsis thaliana infected with M. incognita at 7 (B) and 21 (C) DAI. Left panel, fluorescence images of Alexa-488 (green), and DAPI-stained nuclei (blue) appear paired with corresponding DIC overlays on the right panel. n, nematode; * giant cell. Bars = 10 µm. (TIF) [file pone.0064586.s004.tif]
